# Ilio-psoas impingement with a dual-mobility liner: an original case report and review of literature

**DOI:** 10.1051/sicotj/2020025

**Published:** 2020-07-10

**Authors:** Michel Henri Fessy, Louis Riglet, Laure-Lise Gras, Hadrien Neyra, Jean-Baptiste Pialat, Anthony Viste

**Affiliations:** 1 Hospices Civils de Lyon, Hôpital Lyon Sud, Service de Chirurgie Orthopédique 165 Chemin du Grand Revoyet 69495 Pierre Benite Cedex France; 2 Univ de Lyon, Université Claude Bernard Lyon 1, Univ Gustave Eiffel, IFSTTAR, UMRT_9406, Laboratoire de Biomécanique et Mécanique des Chocs 69622 Lyon France; 3 Hospices Civils de Lyon, Hôpital Lyon Sud, Service de Radiologie 165 Chemin du Grand Revoyet 69495 Pierre Benite Cedex France

**Keywords:** Iliopsoas impingement, Dual mobility, Liner, Total hip arthroplasty

## Abstract

Ilio-psoas impingement after total hip arthroplasty often occurs with the metallic rim of the acetabular cup. The main causes are poor cup anteversion or anterior wall defect. We firstly report here the case of a patient complaining of iliopsoas impingement due to contact with the liner of a dual-mobility device. Ultrasonography and Computed Tomographic scan clearly showed the direct mechanical contact of the dual-mobility liner with the iliopsoas tendon.

## Introduction

Iliopsoas impingement was originally described after total hip arthroplasty by impingement with the metallic rim of a cup [1, 2], metallic heads (metal-on-metal bearings, hip resurfacing or hemiarthroplasty), screws [2], or pegs [3]. Poor anteversion of the cup or anterior cup prominence [4] is one of the main causes leading to iliopsoas impingement.

Large diameter mobile liner of a dual-mobility device was hypothesized *in vitro* as a new cause of iliopsoas impingement [5–7].

## Case presentation

**Figure 1:**
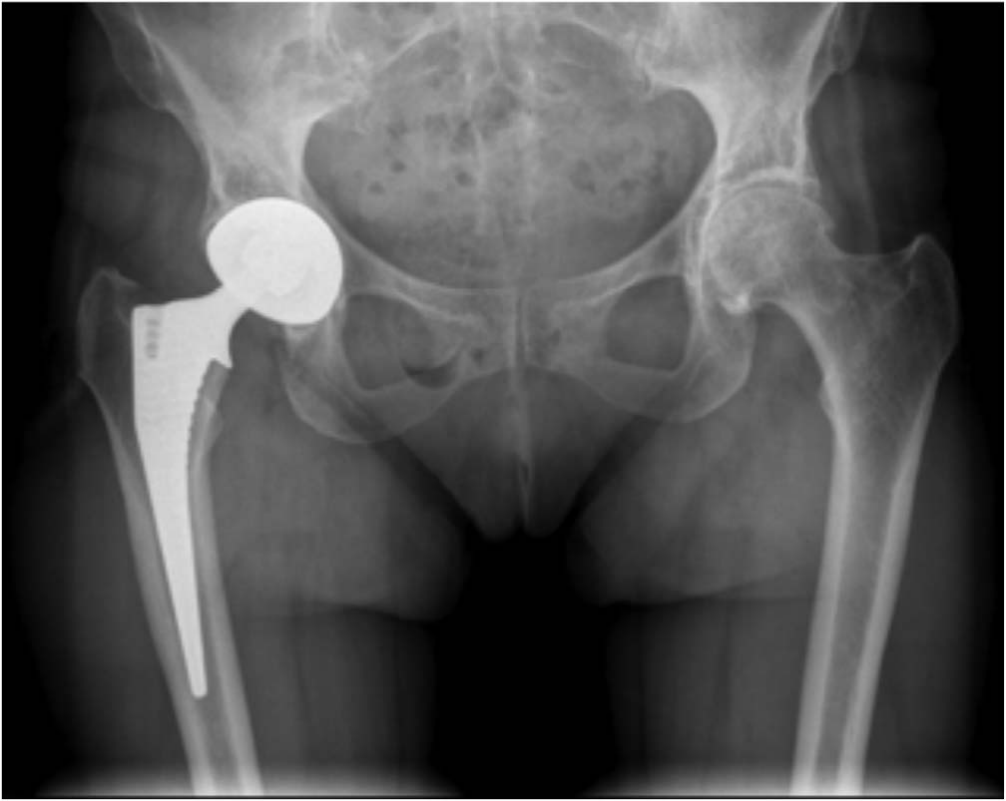
Anteroposterior radiograph of the pelvis showing the right Total Hip Arthroplasty.

In 2015, a 67-year-old woman complained of hip pain for 5 years. She underwent a primary THA using an anterior approach. A dual mobility device (Tornier, France) was implanted: cup size was 46 mm, head size was 22 mm and a lateralized Meije stem (Tornier) (Figure 1).

She was doing well during one year after surgery.

One year later, she complained of anterior hip pain during flexion, difficulties to get into her car, and extreme pain when climbing stairs. Physical examination was typical of iliopsoas impingement: groin pain with resisted hip flexion and resisted straight-leg raise. In order to confirm our hypothesis, we performed an ultrasonography and an arthro-CT-scan.

**Figure 2:**
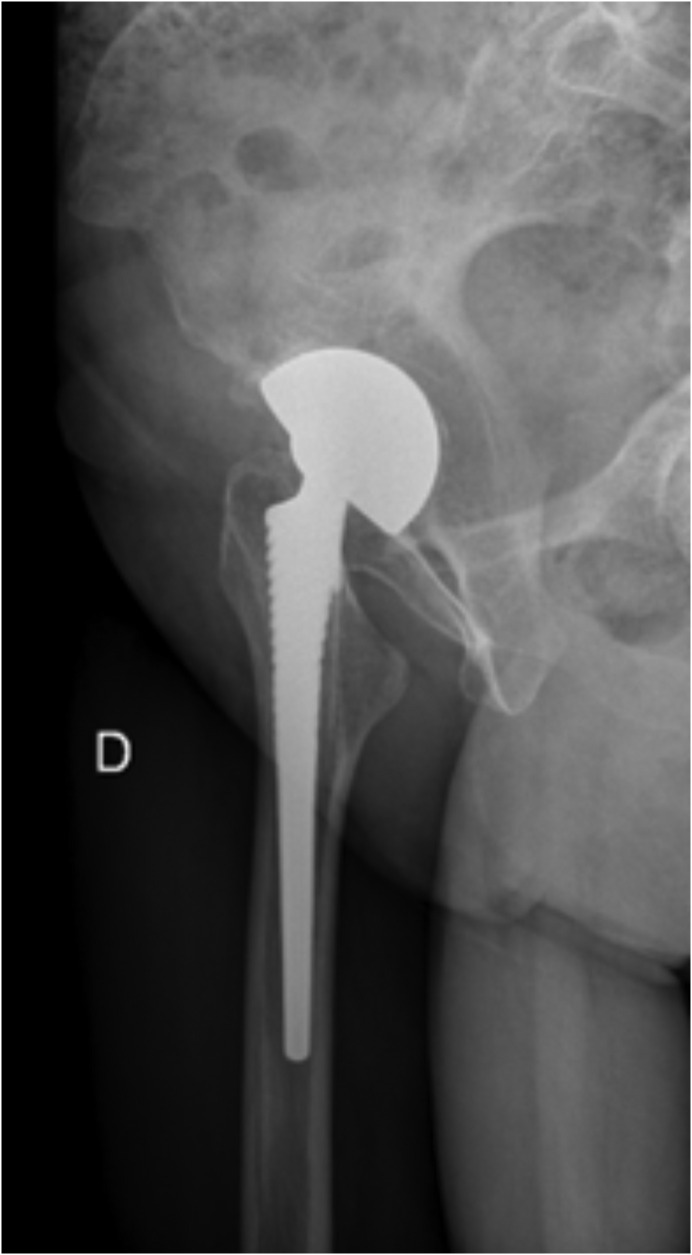
Lateral radiograph of the right total hip arthroplasty.

Ultrasonography found soft-tissue impingement of the large diameter mobile liner with the iliopsoas tendon (Figure 2). Pain was felt by the patient when the US probe was placed on the iliopsoas tendon at the area of contact with the polyethylene liner.

**Figure 3:**
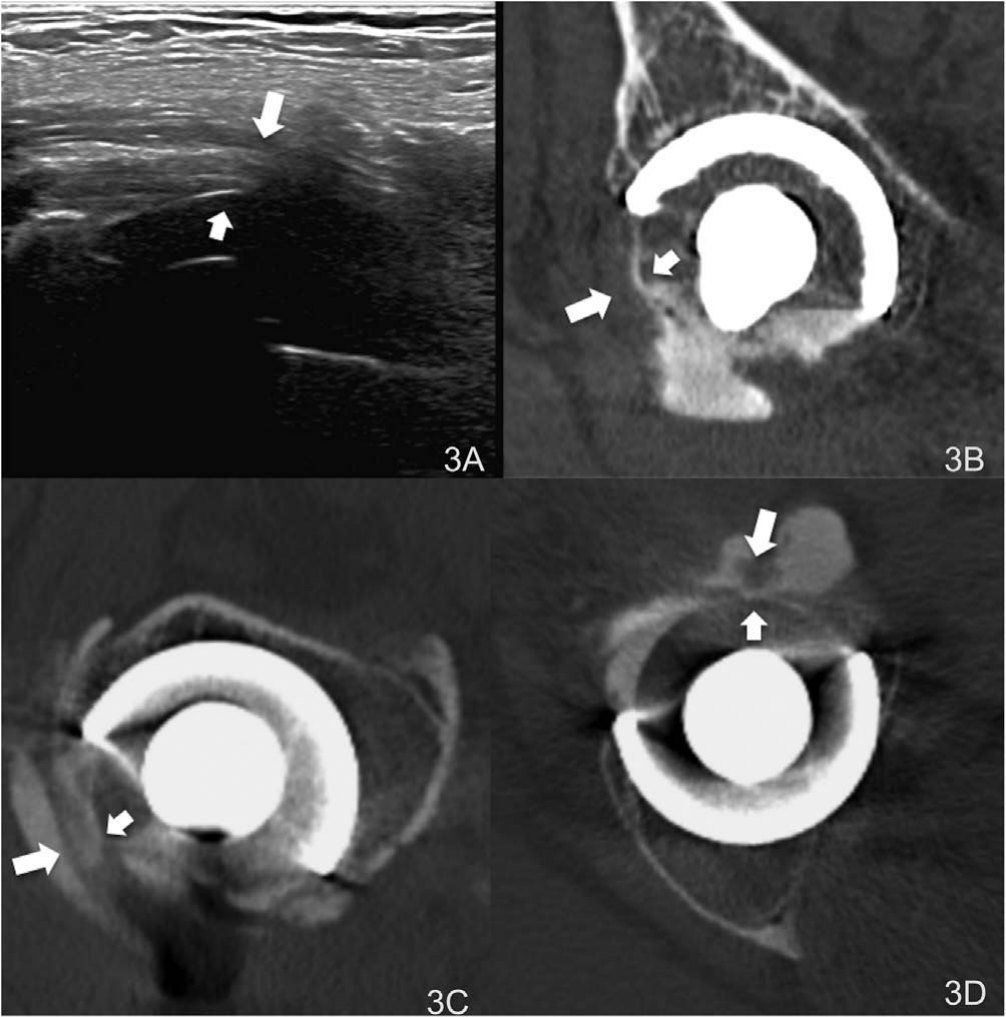
(A) Ultrasonography showing the DM liner (small arrow) and the iliopsoas tendon (big arrow). Arthro-CT-scan: impingement between the DM liner (small arrows and iliopsoas tendon (big arrow) showed in the sagittal (B), coronal oblique (C) and axial plane (D).

An arthro-CT scan was performed and showed that the liner impinged with the iliopsoas tendon (Figure 3). Cup is well anteversed, small size, and well centered. Therefore, nothing about cup orientation could lead to iliopsoas impingement.

## Discussion

To the best of our knowledge, this case report is the first to report an *in vivo* impingement between the liner of a DM device and the iliopsoas tendon. Moreover, we originally describe the ability to diagnose this new phenomenon using ultrasonography and arthro-CT-scan.

Iliopsoas impingement was often described with the metallic rim of the cup [4, 8] or screws [2] and pegs [3]. But this is the very first description in literature of iliopsoas impingement with the mobile liner of a DM device. This phenomenon is very rare as it was never reported before in large cohort of patients with DM cups [9].

Firstly, in 2015, Varadarajan et al. [7] stated that the two main drawbacks with DM were intra-prosthetic dislocation and anterior hip pain. Following the study of Cobb et al. [10], they theorized that reducing the liner profile of a DM device would decrease the rate of anterior hip pain by impingement with the iliopsoas tendon.

In 2016, Nebergall et al. [5] assessed the damage of dual-mobility liner rims in 15 retrieved polyethylenes. In retrieved liners, they found liner rim deformation at a mean 3-year follow-up and they hypothesized that it was due to inhibition of the liner mobility. In a cadaver model, they reported that liner motion was disturbed because of impingement with the iliopsoas tendon.

Then, in 2018, Zumbrunn et al. [6] performed a finite element analysis showing that an anatomical contoured DM liner would avoid the impingement with the iliopsoas tendon.

All these preliminary studies are in favor of a possible impingement between the PE liner of a DM device and the iliopsoas tendon.

## Conclusion

This case report dealt with an original case of impingement between a DM liner and iliopsoas tendon. Further studies will be conducted to study the in vivo motion of the liner during daily tasks. Revision of patients complaining of anterior hip pain would help us to confirm our hypothesis.

## Conflict of interest

The authors have no conflict of interest in relation to this paper. AV is consultant for Serf and Smith & Nephew and MHF receives royalties from Serf and DePuy outside the submitted work.

## Funding

No funding was received for the submitted work.
